# Does a Small Body Have a Negative Impact on Minimally Invasive Mitral Valve Surgery?

**DOI:** 10.3389/fsurg.2021.746302

**Published:** 2022-01-31

**Authors:** Tomonori Shirasaka, Shingo Kunioka, Yuta Kikuchi, Natsuya Isikawa, Hirotsugu Kanda, Hiroyuki Kamiya

**Affiliations:** ^1^Department of Cardiac Surgery, Asahikawa Medical University, Asahikawa, Japan; ^2^Department of Anesthesiology and Critical Care Medicine, Asahikawa Medical University, Asahikawa, Japan

**Keywords:** mitral valve surgery, minimally invasive cardiac surgery, physique, small body, safety

## Abstract

**Backgrounds:**

Minimally invasive mitral valve surgery (MIMVS) in patients with a small body presents surgeons with a technically difficult surgical maneuver. We hypothesized that physique might negatively influence the safety and technical complexity of MIMVS.

**Methods:**

One hundred and twenty-one patients underwent MIMVS in our institution between May 2014 and April 2020. These patients were categorized into two groups. The first group was the small physique group (*n* = 20) consisting of patients with a stature <150 cm. The second group was the normal physique group (*n* = 101) consisting of patients with a stature >150 cm. The primary endpoint was freedom from death and major adverse cardiovascular and cerebrovascular events (MACCE). The secondary endpoint was freedom from moderate or severe mitral regurgitation.

**Results:**

Cardiopulmonary bypass time (130 ± 29 vs. 156 ± 55 min, *p* = 0.02) and aortic cross-clamp time (75 ± 27 vs. 95 ± 39 min, *p* = 0.03) were significantly shorter in the small physique group. Both in the early and midterm periods, there was no significant difference in the mortality (early, 5.0 vs. 1.0%, *p* = 0.30. midterm, 5.0 vs. 1.0%, *p* = 0.09), MACCE (early, 5.0 vs. 6.9%, *p* = 0.65. midterm, 5.0 vs. 5.9%, *p* = 0.93) and the residual MR (early, 0 vs. 1.0%, *p* = 0.66. midterm, 5.0 vs. 4.9%, *p* = 0.93) between the two groups.

**Conclusions:**

Small physique is not a hurdle for MIMVS in terms of the safety of the operation.

## Introduction

Previously, the primary candidates for minimally invasive cardiac surgery (MICS) were young patients who were socially active and had low risk and who expected excellent cosmesis (1). However, the indication for this method has recently expanded to relatively elderly people expecting faster recovery brought about by the preservation of their sternum (2). In the future, other good candidates for MICS will be children or adolescents who are seeking a fast return to social activities and better cosmesis.

Preliminary reports in relation to MICS in congenital cardiac surgery have focused on the clinical success of the operation for atrial septal defect (ASD), ventricular septal defect (VSD), and atrioventricular septal defect *via* right thoracotomy (3, 4). For example, Yoshimura et al. reported excellent operative results for patients with ASD (mean age 7 years, mean body weight 23.9 kg). Mishaly et al. performed ASD or VSD closure and mitral valve repair on young patients, including 60 infants (mean age 9 years, mean body weight 20 kg), and concluded that the establishment of cardiopulmonary bypass (CPB) or the maintenance of good exposure in congenital cardiac surgery *via* right thoracotomy could be achieved in children with a body weight >9 kg.

One of the biggest concerns with MICS in patients with a small body is leg ischemia (5) due to the relatively small vascular diameter required for cannulation and the likelihood of spasticity, especially in young patients. Another concern is the technical difficulty in performing surgical maneuvers in a small pleural cavity (6). These concerns might be the primary reasons for the delay in the introduction of recent technological advances in MICS for congenital heart surgery.

Historically, among all variables with an influence on surgical outcome, body weight and body mass index or body surface area (BSA) have also been investigated (7, 8) to determine whether they significantly influence the outcome. However, in previously published reports, full sternotomy was performed in all documented cases, and none of the studies provided information on the impact of quantitative preoperative variables, such as body height, vascular diameter for cannulation, and the vertical or cross diameter of the pleural cavity on the operative results. Thus, elucidation of the negative factors for the completion of MICS would be of great value.

In this study, the patients who underwent mitral valve surgery were categorized into two groups using the MICS approach (MIMVS), namely, patients with a small physique and those with a normal physique, and the patient characteristics and clinical outcomes between the two groups were compared to elucidate the validity of MIMVS for individuals with a small body.

## Methods

The institutional review board of Asahikawa Medical University Hospital approved this retrospective study and waived the need for written patient consent (IRB number: 19083).

### Patient Demographics

**Figure 1:**
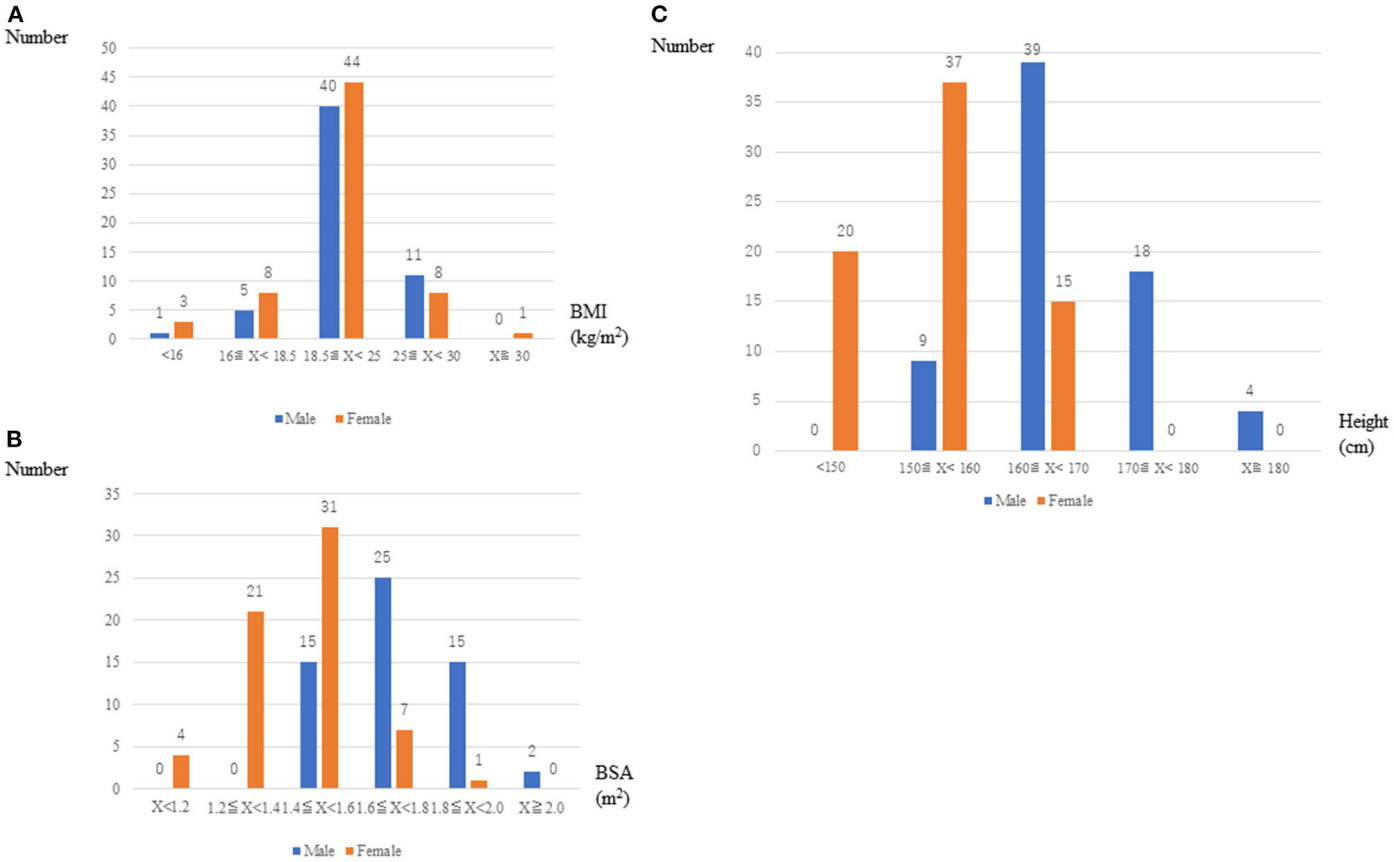
Distribution of BMI, BSA, and height in male and female patients.

A total of 121 patients underwent MIMVS in our institution between May 2014 and April 2020. Figure 1 shows the distribution of body mass index [BMI, 1-(A)], body surface area [BSA, 1-(B)] and height [1-(C)] in these patients. Mean BMI, BSA, and height in male/ female were 22.5 ± 3.1/21.2 ± 3.6 kg/m^2^, 1.7 ± 0.2/1.5 ± 0.2 m^2^, and 167 ± 7.2/153 ± 8.3 cm, respectively.

We categorized these patients into two groups: the small physique group (*n* = 20), which consisted of patients with a stature <150 cm, and the normal physique group (*n* = 101), which consisted of patients with a stature >150 cm.

Table 1 presents the patient demographics of the two groups. A significant difference in age (79 ± 7 vs. 62 ± 15 years, *p* < 0.001), gender (*p* < 0.001), height (143 ± 4.5 vs. 163 ± 8 cm, *p* < 0.001), body weight (42 ± 7.5 vs. 59 ± 10.7 kg, *p* < 0.001), BSA (1.3 ± 0.1 vs. 1.6 ± 0.1, *p* < 0.001), and the diastolic or systolic dimensions of the left ventricle (LVDd, 49 ± 5 vs. 54 ± 8.0 mm, *p* = 0.01. LVDs, 31 ± 6 vs. 35 ± 8.2 mm, *p* = 0.02) was observed between these two groups. Preoperative computed tomography revealed a significant difference in the axial length of the pleural cavity between the two groups (220 ± 10.6 vs. 242 ± 21.7 mm, *p* < 0.001) but none in the vertebra–sternum distance (97 ± 11.7 vs. 102 ± 196 mm, *p* = 0.21) or diameter of the femoral artery (10 ± 1.6 vs. 11 ± 2.0 mm, *p* = 0.15). Our exclusion criteria for MIMVS are as follows: 1. The existence of calcified or atherothrombotic ascending aorta. 2. The existence of significant coronary artery disease. 3. Severe mitral annular calcification. 4. Very obese patient (BMI ≧ 35) or critical emaciation (BMI ≦ 15). 5. Poor femoral or iliac access.

**Table 1:** Patient demographics.

**Variable**	**Small physique** **(*n* = 20)**	**Normal physique** **(*n* = 101)**	***p*-value**
Age, y	79 ± 7	62 ± 15	<0.001^*^
Gender, male	0	57	<0.001^*^
Height, cm	143 ± 4.5	163 ± 8	<0.001^*^
Body weight, kg	42 ± 7.5	59 ± 10.7	<0.001^*^
BMI, kg/ m^2^	20.6 ± 3.3	22.0 ± 3.4	0.22
BSA, m^2^	1.3 ± 0.1	1.6 ± 0.1	<0.001^*^
NYHA class			
0/I/II/III/IV	5/10/5/0	37/39/20/5	–
≧III	5	25	0.98
Preoperative TTE			
LVDd, mm	49 ± 5	54 ± 8.0	0.01^*^
LVDs, mm	31 ± 6	35 ± 8.2	0.02^*^
EF, %	66 ± 8.1	63 ± 10.2	0.23
MR grade			
0/I/II/III/IV	0/1/0/0/19	0/3/1/4/93	
AR grade			
0/I/II/III/IV	4/7/7/1/0	59/19/23/0/0	
Etiology of mitral valve			
Degenerative	18	88	0.73
MS, rheumatic	1	6	0.75
IE	1	7	0.85
Detail of MR	19	101	
Type I/II/III	3/15/1	14/76/5	
Atrial functional MR	3	14	0.89
Preoperative CT			
Vertebra–sternum distance, mm	97 ± 11.7	102 ± 19.6	0.21
Axial length of the pleural cavity, mm	220 ± 10.6	242 ± 21.7	<0.001^*^
Diameter of the femoral artery, mm	10 ± 1.6	11 ± 2.0	0.15

*BMI, body mass index; BSA, body surface area; CT, computed tomography; EF, ejection fraction; MR, mitral regurgitation; NYHA, New York Heart Association; TTE, transesophageal echocardiography*.

* *Means the existence of significant difference of p-value*.

### Operative Procedure of MICS Mitral Surgery

In this study, 2 surgeons performed MIMVS. In our unit, MIMVS was performed under direct vision with the assistance of thoracoscope routinely for the surgery. Femoral cannulation was performed in surgical cut down technique using a one-stage arterial cannula [PCKC-A 16 French (Fr), 18 Fr, Toyobo, Osaka, Japan] and a single venous cannula (QuickDraw, 25 Fr, Edwards Lifesciences, Irvine, CA, USA) or a two-stage venous cannula (RAP FV cannula 22–25 Fr, LivaNova, Milan, Italy) inserted through the right atrium into the superior vena cava. During CPB, carbon dioxide insufflation of 3 L/min was employed for de-airing of the heart. A 5- to 7-cm right thoracotomy was performed *via* the fourth intercostal space. After establishing a full CPB flow, pericardiotomy was performed. Body temperature was decreased to mild hypothermia of 30–32°C. After aortic cross-clamp using a Cygnet flexible clamp (Vitalitec International, Inc., Plymouth, MA, USA), initial cardioplegia was delivered in an antegrade fashion, followed by left atriotomy with intermittent delivery of blood cardioplegia performed every 30 min.

The left atrium (LA) was exposed by the gentle retraction of the ascending aorta by LA retractor (Adams-Yozu Mini-Valve System, Geister, Tuttlingen, Germany). After mitral valve surgery was completed, LA was closed in a single-layer fashion followed by declamp without terminal hot-shot of cardioplegia. RV pacing lead was placed before routinely releasing the cross-clamp.

### Postoperative Anticoagulation

Postoperative oral anticoagulation was administered. Irrespective of the rhythm pattern, warfarin was routinely administered to all patients, and the prothrombin time–international normalized ratio was controlled between 1.8 and 3.0. If sinus rhythm was stable for longer than 3 months, warfarin therapy was discontinued, except in cases of mitral valve replacement with a mechanical valve.

### Endpoint

The primary endpoint was freedom from death and major cardiac and cerebrovascular event (MACCE). The secondary endpoint was freedom from moderate or severe mitral regurgitation.

### Statistical Analysis

Preoperative, intraoperative, and postoperative data were recorded and retrospectively reviewed. Continuous variables were expressed as mean ± standard deviation, and categorical variables were presented as either absolute numbers or percentages.

Baseline patient characteristics, operative data, postoperative complications, and outcome rates were compared using Pearson's chi-squared test or Fisher's exact test for categorical variables or Student's *t*-test or the Wilcoxon rank-sum test for continuous variables. The significance level was set at *p* < 0.05. For the time-to-event analysis, we employed log-rank tests and the Kaplan–Meier method. The Kaplan–Meier method was employed to demonstrate overall survival, freedom from MACCE and freedom from significant MR defined as moderate or severe MR. Statistical analyses were conducted using STATA version 15.1 (StataCorp LP, College Station, TX, USA).

## Results

Operative time, cardiopulmonary time, and aortic cross-clamp time were significantly shorter in the small physique group than in the normal physique group (operative time, 177 ± 45 vs. 201 ± 60 min, *p* = 0.02; cardiopulmonary time, 130 ± 29 vs. 156 ± 55 min, *p* = 0.02; aortic cross-clamp time, 75 ± 27 vs. 95 ± 39 min, *p* = 0.03; Table 2), despite the fact that the percentages of isolated mitral valve surgery in both groups were not significantly different (*p* = 0.42; Table 2).

**Table 2:** The details of operative procedures.

**Variable**	**Small physique** **(*n* = 20)**	**Normal physique** **(*n* = 101)**	***p*-value**
Operative time	177 ± 45	210 ± 60	0.02*
CPB time	130 ± 29	156 ± 55	0.02*
ACC time	75 ± 27	95 ± 39	0.03*
Second run of CPB	0	6	0.54
Operative procedures				
Mitral valve	20	101	–
Isolated mitral valve surgery	16	69	0.42
Annuloplasty	18	88	0.72
Ring	9	41	0.81
Band	9	47	0.90
Valve repair	16	69	0.98
Triangular resection	4	28	0.59
Folding plasty	5	22	0.77
Indentation closure	2	20	0.52
Artificial chordae	3	23	0.56
Chordal transplantation	0	3	0.44
Central edge to edge	0	1	0.66
Commissural closure	3	7	0.37
Patch augmentation	0	3	0.44
Valve replacement	2	10	0.98
Tricuspid valve repair	4	32	0.42
Surgical maze procedure	2	28	0.15

*ACC, aortic cross-clamp; CPB, cardiopulmonary bypass*.

In relation to the details of the operative procedures, no significant difference was observed between the group in the use of materials for mitral annuloplasty (*p* = 0.72), frequency of valve repair (*p* = 0.98), concomitant tricuspid valve repair (*p* = 0.42), and antiarrhythmic operation (*p* = 0.15) (Table 2).

In the early postoperative periods, one patient in poor preoperative condition both in the small and normal physique groups died, respectively (30 days mortality, 5.0 vs. 1.0%, *p* = 0.30). There was no significant difference in the rate of MACCE and significant residual MR between two groups (MACCE, 5.0 vs. 6.9%, *p* = 0.65. ≧Moderate MR, 0 vs. 1.0%, *p* = 0.66; Table 3).

**Table 3:** Operative outcomes in the early and midterm periods.

**Variable (percentage)**	**Small physique** **(*n* = 20)**	**Normal physique** **(*n* = 101)**	***p*-value**
30-day mortality	1 (5.0)	1 (1.0)	0.30
In-hospital mortality	1 (5.0)	1 (1.0)	0.30
MACCE (including death)	1 (5.0)	7 (6.9)	0.65
Reexploration for bleeding	1 (5.0)	2 (2.0)	0.42
Reexpansion pulmonary edema	0	0	–
Pulmonary herniation	0	2 (2.0)	0.53
Stroke	0	0	–
SSI	0	0	–
Reoperation within 12 months	0	3 (3.0)	0.44
Readmission due to HF	0	0	–
Postoperative TTE at discharge			
≧Moderate MR	0	1 (1.0)	0.66
Mean PG bw LA-LV, mm Hg	2.5 ± 1.1	2.8 ± 1.4	0.38
Latest postoperative TTE			
Follow-up periods, years	2.1 ± 1.4	2.9 ± 1.8	0.09
≧Moderate MR	1 (5.0)	5 (4.9)	0.93
All-cause death	1 (5.0)	1 (1.0)	0.09
MACCE (including death)	1 (5.0)	6 (5.9)	0.93
Reoperation	0	5 (4.9)	0.31

*SSI, surgical site infection; HF, heart failure; MR, mitral regurgitation; TTE, transesophageal echocardiography*.

**Figure 2:**
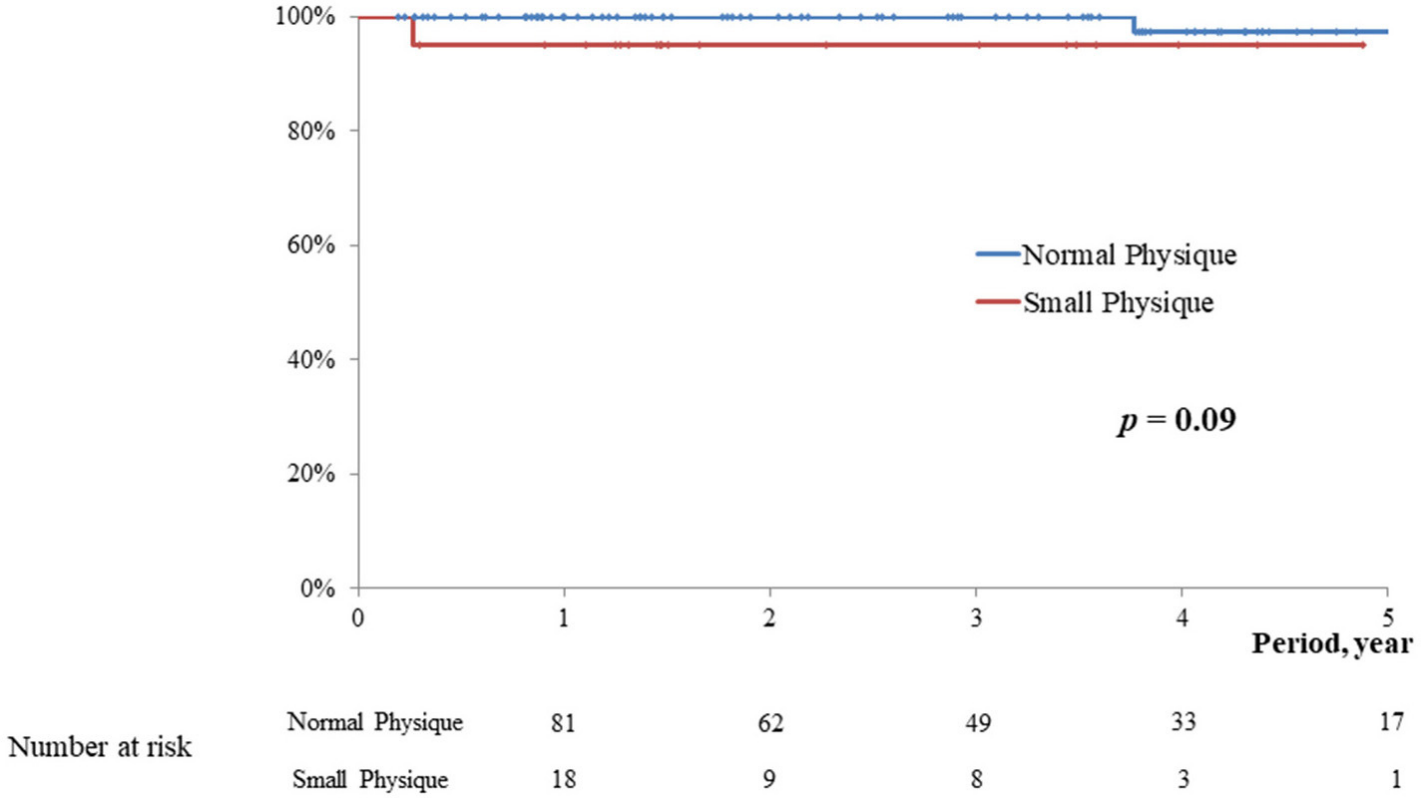
Survival.

**Figure 3:**
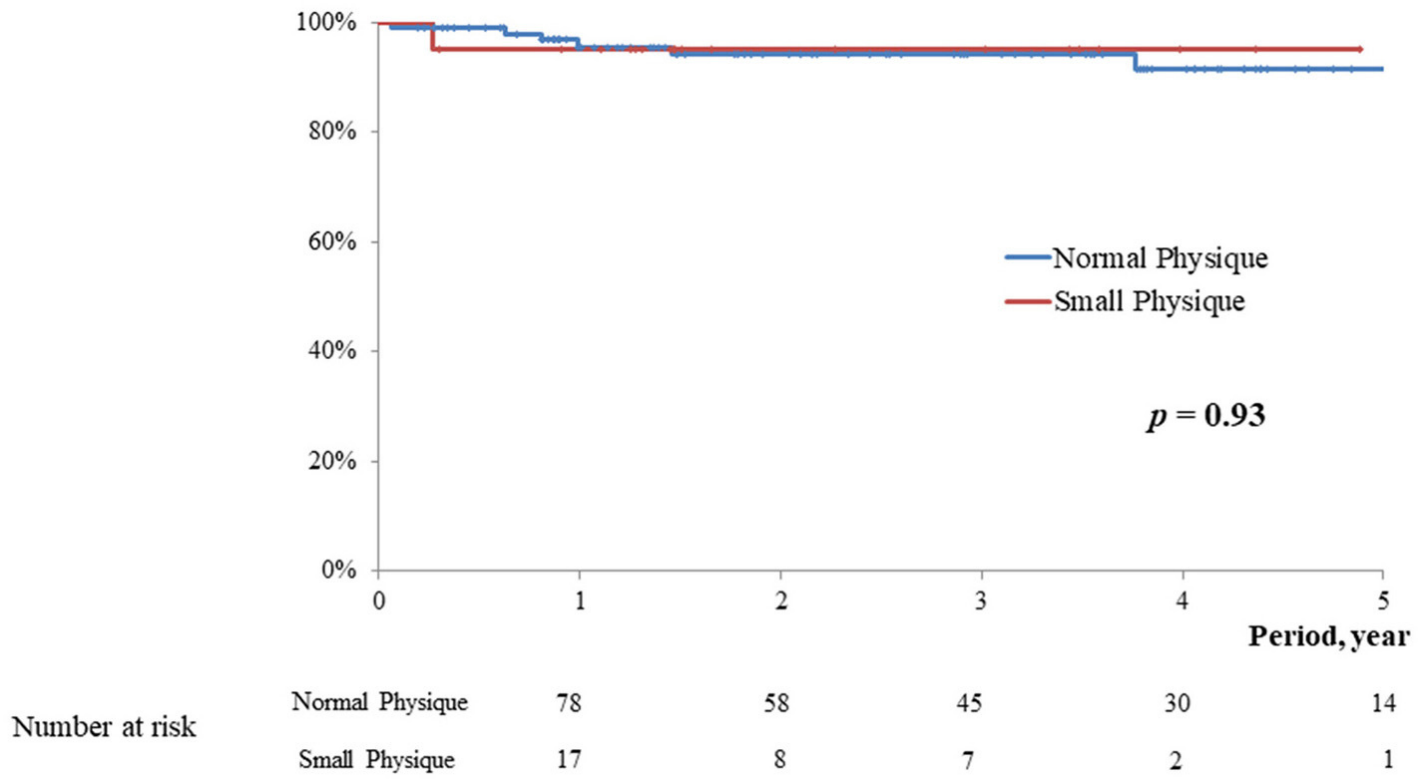
Freedom from MACCE.

**Figure 4:**
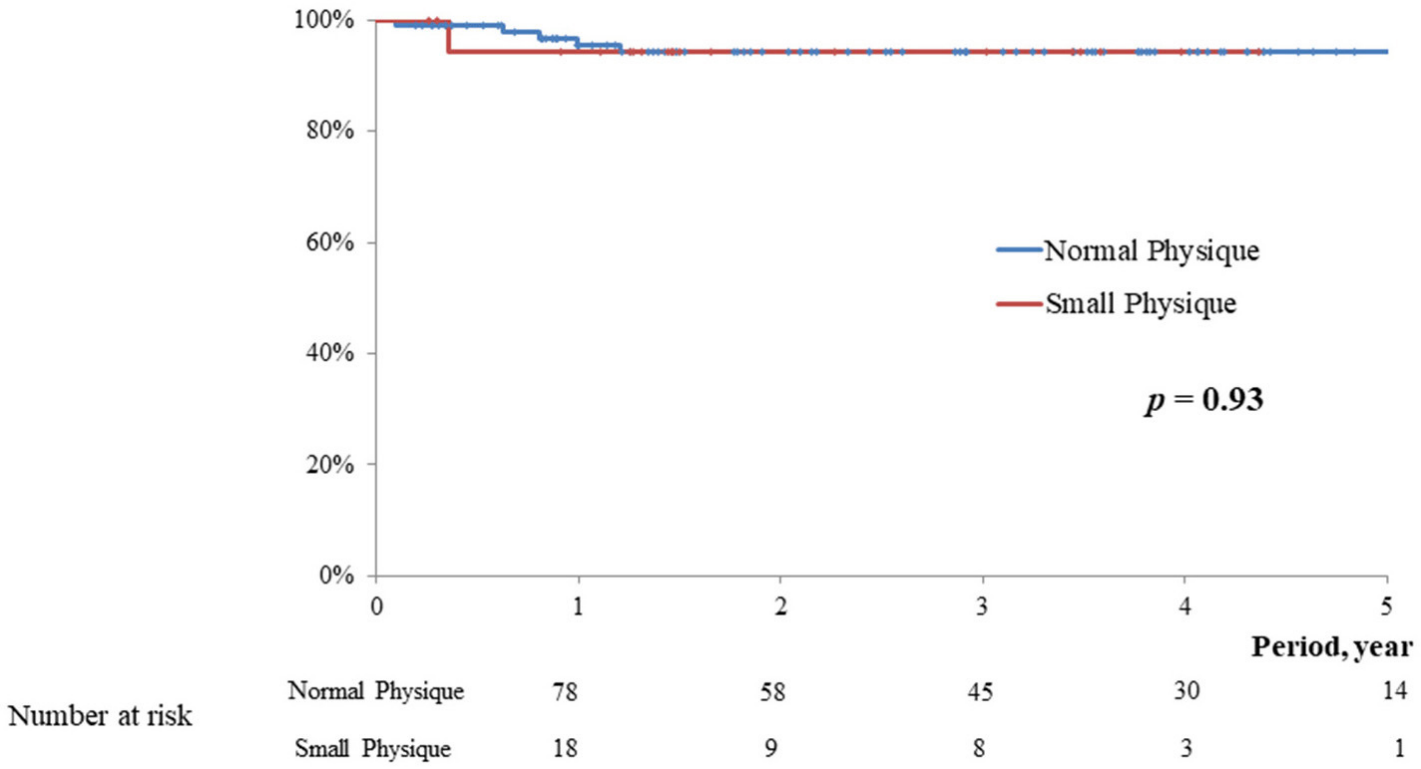
Freedom from ≧ moderate MR.

The postoperative follow-up periods were 2.1 ± 1.4 vs. 2.9 ± 1.8 year, respectively (*p* = 0.09). In the midterm, no significant difference was observed in the rate of overall death (5.0 vs. 1.0%, *p* = 0.09), MACCE (5.0 vs. 5.9%, *p* = 0.93), postoperative significant mitral regurgitation (5.0 vs. 4.9%, *p* = 0.93), and reoperation (0 vs. 4.9%, *p* = 0.31) between the two groups. Kaplan-Meyer curve in relation to overall survival, freedom from MACCE, and freedom from significant MR were not significantly different between the two groups (*p* = 0.09, 0.93, and 0.93, respectively; Figures 2–4).

## Discussion

In general, MICS requires performing surgical maneuvers using long-shafted instruments in small spaces, which is not easy due to the technical aspects. Therefore, appropriate patient selection is important, taking into consideration the safety of the procedure, the surgeon's level of comfort performing the surgical maneuvers, and whether the clinical outcome is promising and not compromised as compared with conventional open-heart surgery.

We hypothesized that a small physique might have an influence on the safety or technical complexity of MICS. However, the findings of this study suggest that CPB and aortic cross-clamp time as well as operative time in the small physique group was not longer than that in the normal physique group, although 75% in the small physique group underwent isolated MVP while 64% in the normal physique group and this might be related to the result that CPB and cross clamp time were shorter in the small physique group.

The definition of small stature may vary based on the patient's race. Short stature is defined as an adult height greater than two standard deviations below the mean for age and gender, which corresponds to the shortest 2.3% of individuals (9). Typically, in developed countries, this includes adult men shorter than 158 cm and adult women shorter than 149 cm. A recent national health and nutrition survey in Japan (10) revealed that the mean height of adult men and women in Japan was 168 ± 7.1 and 154.5 ± 7.0 cm, respectively. Taking this into consideration, the definition of small physique in this study was defined as a height <150 cm.

Historically, researchers have discussed how the difference in body size influences the outcomes of cardiac surgery. In their multiple regression analysis, Tarui et al. found significant associations among BMI, cannula size, and operation time and an increase in postoperative creatinine phosphorus kinase (11). Several reports have elucidated the strong association between large BSA or BMI (7, 8, 12) and the inferiority in clinical outcome, indicating that obese individuals typically have multicomorbidities, such as diabetes, hypertension, and impaired respiratory function. However, all of these studies precluded only conventional cardiac surgery *via* full sternotomy and did not discuss the influence of this factor on the technical complexity in MICS. To the best of our knowledge, this is the first report elucidating whether there is a significant association between small body size and clinical outcome in MIMVS.

Stable perfusion is important in open-heart surgery, especially in MICS. The most typical venous drainage setup in MICS is femoral vein cannulation (13). Poor venous drainage leads to a bad exposure of the mitral valve, making the surgery itself uncomfortable. In addition, venous back pressure results in the washout of cardioplegia, diminishing its cardioprotective effects, which may result in perioperative ventricular dysfunction (14). In the present study, we used low-profile venous cannulas (22 or 25 Fr); thus, we rarely experienced any difficulty in cannulation or in setting the position of the tip of the venous cannula appropriate to the drain under the guidance of intraoperative transesophageal echocardiography, even in patients with small physique. With regard to arterial cannulation in MICS, the common femoral artery (CFA) is the most commonly adopted site. The CFA diameter steadily increases throughout life, and it is significantly correlated with height, weight, and BSA. In addition, the CFA diameter is larger in men than in women (15). Limited to our study, all the patents in the small physique group were elderly women, and the mean diameter of the femoral artery in the two groups was not significantly different from that in the normal physique group. This feature of the current study might have influenced the outcome, which was different from our hypothesis.

As an indicator for the estimation of the actual surgical space in MICS mitral value surgery, the distance between the vertebrae and sternum is regarded as quite important for determining whether the MICS approach is optimal. This distance should preferably be 80 mm or greater for direct vision MICS (6). In fact, in our small physique group, this variable was beyond this threshold. Moreover, none of the patients in the small physique group demonstrated a funnel chest or thorax deformity.

The finding in this study, namely, that physique has no impact on the surgical outcome in MIMVS, contributes to the extension of the indication for the MICS approach in mitral valve surgery to small elderly people or young patients in adolescence who expect a quick recovery *via* preservation of their sternum and subsequent fast return to their daily lives.

### Study Limitations

This study had some limitations. Namely, this was a retrospective study, and the sample size of the two groups was quite small. Patients' selection referred by our exclusion criteria was unavoidable especially early in the series in terms of patient safety.

In this study, small physique group was constituted only by women unexpectedly. It was because that we set the threshold of statue for small physique as 150 cm referring to the national database about physique in Japanese people. We intentionally set only a single parameter (height) as an influencer of the body shape itself on surgical outcome. However, various factors such as BSA or BMI may influence to the clinical outcome of surgery in real practice. Actually, the number of very obese patient whose BMI over 30 was only 1, and that of very slim or skinny patients whose BMI under 16 was 4 in this series (Figure 1). Similarly, the number of the patient whose BSA was over 2.0 was only 2 people, and whose BSA under 1.2 was only 4 in this series (Figure 1). Moreover, all of them got well soon after MICS uneventfully. So, it is difficult to elucidate the power of very high or low BMI on the clinical outcome in MICS in this study.

Despite these limitations, the strengths of this study include the quite conclusive findings, with complete clinical follow-up examinations performed for all patients, including late echocardiographic follow-up findings.

Our results reconfirm the feasibility of the MICS approach in patients with a small body.

In conclusion, a small physique is not a contraindication for MIMVS.

## Data Availability Statement

The raw data supporting the conclusions of this article will be made available by the authors, without undue reservation.

## Ethics Statement

The studies involving human participants were reviewed and approved by the Institutional Review Board of Asahikawa Medical University Hospital. The patients/participants provided their written informed consent to participate in this study.

## Author Contributions

All authors listed have made a substantial, direct, and intellectual contribution to the work and approved it for publication.

## Conflict of Interest

The authors declare that the research was conducted in the absence of any commercial or financial relationships that could be construed as a potential conflict of interest.

## Publisher's Note

All claims expressed in this article are solely those of the authors and do not necessarily represent those of their affiliated organizations, or those of the publisher, the editors and the reviewers. Any product that may be evaluated in this article, or claim that may be made by its manufacturer, is not guaranteed or endorsed by the publisher.

## References

[1] Sakaguchi T. Minimally invasive mitral valve surgery through a right mini-thoracotomy. Gen Thorac Cardiovas. (2016) 64:699–706. 10.1007/s11748-016-0713-5 27638268

[2] Iribarne A Easterwood R Russo MJ Chan EY Smith CR Argenziano M. Comparative effectiveness of minimally invasive versus traditional sternotomy mitral valve surgery in elderly patients. J Thorac Cardiovasc Surg. (2012) 143:S86–90. 10.1016/j.jtcvs.2011.10.090 22423605

[3] Yoshimura N Yamaguchi M Oshima Y Oka S Ootaki Y Yoshida M. Repair of Atrial septal defect through a right posterolateral thoracotomy: a cosmetic approach for female patients. Ann Thorac Surg. (2001) 72:2103–5. 10.1016/S0003-4975(01)03086-7 11789801

[4] Mishaly D Ghosh P Preisman S. Minimally invasive congenital cardiac surgery through right anterior minithoracotomy approach. Ann Thorac Surg. (2008) 85:831–5. 10.1016/j.athoracsur.2007.11.068 18291151

[5] Toya T Fujita T Fukushima S Shimahara Y Kume Y Yamashita K. Efficacy of regional saturation of oxygen monitor using near-infrared spectroscopy for lower limb ischemia during minimally invasive cardiac surgery. J Artif Organs. (2018) 21:420–6. 10.1007/s10047-018-1057-y 29938392

[6] Ito T. Minimally invasive mitral valve surgery through right mini-thoracotomy: recommendations for good exposure, stable cardiopulmonary bypass, and secure myocardial protection. Gen Thorac Cardiovasc Surg. (2015) 63:371–8. 10.1007/s11748-015-0541-z 25840800

[7] Schwann TA Habib RH Zacharias A Parenteau GL Riordan CJ Durham SJ. Effects of body size on operative, intermediate, and long-term outcomes after coronary artery bypass operation. Ann Thorac Surg. (2001) 71:521–31. 10.1016/S0003-4975(00)02038-5 11235700

[8] Lio A Emanuele Bovio E Nicolò F Saitto G Scafuri A Bassano C. Influence of body mass index on outcomes of patients undergoing surgery for acute aortic dissection: a propensity-matched analysis. Tex Heart Inst J. (2019) 46:7–13. 10.14503/THIJ-17-6365 30833831 PMC6378991

[9] Pedicelli S Peschiaroli E Violi E Cianfarani S. Controversies in the definition and treatment of idiopathic short stature (ISS). J Clin Res Ped Endo. (2009) 1:105–15. 10.4008/jcrpe.v1i3.53 21274395 PMC3005647

[10] The National Health and Nutrition Survey in Japan. (2018). Available online at: http://www.nibiohn.go.jp/eiken/kenkounippon21/en/eiyouchousa/

[11] Tarui T Miyata K Shigematsu S Watanabe G. Risk factors to predict leg ischemia in patients undergoing single femoral artery cannulation in minimally invasive cardiac surgery. Perfusion. (2018) 33:533–7. 10.1177/0267659118768151 29637839

[12] Habib RHD Zacharias A Schwann TA Riordan CJ Durham SJ Shah A. Effects of obesity and small body size on operative and long-term outcomes of coronary artery bypass surgery: a propensity-matched analysis. Ann Thorac Surg. (2005) 79:1976–86. 10.1016/j.athoracsur.2004.11.029 15919295

[13] Lamelas J Aberle C Macias AE Alnajar A. Cannulation strategies for minimally invasive cardiac surgery. Innovations. (2020) 15:261–9. 10.1177/1556984520911917 32437215

[14] Momin AA Toth AJ Gillinov AM Wierup P Mick SL. Exploring ventricular dysfunction and poor venous drainage during robotic mitral valve surgery. J Card Surg. (2020) 35:1253–7. 10.1111/jocs.14574 32333432

[15] Sandgren T Sonesson B Ahlgren AR Toste Länne T. The diameter of the common femoral artery in healthy human: influence of sex, age, and body size. J Vasc Surg. (1999) 29:503–10. 10.1016/S0741-5214(99)70279-X 10069915

